# Recurrence and mortality 1 year after hospital admission for non-fatal self-harm: a nationwide population-based study

**DOI:** 10.1017/S2045796019000039

**Published:** 2019-02-18

**Authors:** A. Vuagnat, F. Jollant, M. Abbar, K. Hawton, C. Quantin

**Affiliations:** 1 Department for Research, Studies, Evaluation and Statistics (DREES), French Health and Social Affairs Ministry, Paris, France; 2 Biostatistics and Bioinformatics (DIM), University Hospital, Dijon & France Bourgogne Franche-Comté University, Dijon, France; 3 Paris-Descartes University, & Sainte-Anne Hospital, Paris, France; 4 McGill group for suicide studies, McGill University, Montréal, Canada; 5 Academic Hospital (CHU) Nîmes, Nîmes, France; 6 Department of Psychiatry, University of Oxford, Warneford Hospital, Oxford, UK; 7 Inserm, CIC 1432, Dijon, France; 8 Dijon University Hospital, Clinical Investigation Center, clinical epidemiology/clinical trials unit, Dijon, France; 9 Biostatistics, Biomathematics, Pharmaco-epidemiology and Infectious Diseases (B2PHI), Inserm, UVSQ, Institut Pasteur, Université Paris-Saclay, Paris, France

**Keywords:** mortality, recurrence, self-harm, suicide

## Abstract

**Aims:**

A large number of people present each day at hospitals for non-fatal deliberate self-harm (DSH). Examination of the short-term risk of non-fatal recurrence and mortality at the national level is of major importance for both individual medical decision-making and global organisation of care.

**Methods:**

Following the almost exhaustive linkage (96%) of two national registries in France covering 45 million inhabitants (i.e. 70% of the whole population), information about hospitalisation for DSH in 2008–2009 and vital status at 1 year was obtained. Individuals who died during the index hospital stay were excluded from analyses.

**Results:**

Over 2 years, 136,451 individuals were hospitalised in medicine or surgery for DSH. The sample comprised 62.8% women, median age 38 in both genders, with two peaks at 16 and 44 years in women, and one peak at 37 years in men. The method used for DSH was drug overdose in 82.1% of cases. Admission to an intensive care unit occurred in 12.9%. Following index hospitalisation, 71.3% returned home and 23.7% were transferred to a psychiatric inpatient care unit. DSH recurrence during the following year occurred in 12.4% of the sample, within the first 6 months in 75.2%, and only once in 74.6%. At 1 year, 2.6% of the sample had died. The overall standardised mortality ratio was 7.5 but reached more than 20 in young adults. The causes were natural causes (35.7%), suicide (34.4%), unspecified cause (17.5%) and accident (12.4%). Most (62.9%) deaths by suicide occurred within the first 6 months following index DSH. Violent means (i.e. not drug overdose) were used in 70% of suicide cases. Concordance between means used for index DSH and for suicide was low (30% overall), except for drug overdose. Main suicide risk factors were older age, being male, use of a violent means at index DSH, index admission to an intensive care unit, a transfer to another medical department or to a psychiatric inpatient unit, and recurrence of DSH. However, these factors had low positive predictive values individually (below 2%).

**Conclusions:**

Non-fatal DSH represent frequent events with a significant risk of short-term recurrence and death from various causes. The first 6 months following hospital discharge appear to be a critical period. Specific short-term aftercare programs targeting all people with a DSH episode have to be developed, along other suicide prevention strategies.

## Introduction

Non-fatal deliberate self-harm (DSH) represents considerable public health issues. The lifetime prevalence of DSH has been estimated at 1.3% of the general population in Europe (Bernal *et al*., 2007). In addition, people who present to hospital for non-fatal DSH are at increased risk of premature death. Studies have robustly shown that these acts are the strongest risk factor for future suicide (Cavanagh *et al*., 2003). A recent meta-analysis based on 40 studies estimated the risk of subsequent suicide death during the following year around 1.6% (CI 1.2–2.4) (Carroll *et al*., 2014). Rates of suicide in those who self-harmed during the previous year are therefore in the range of 100 times the rates in the general population in Europe and North America (Owens *et al*., 2002). However, excess mortality in people who self-harmed is not solely attributable to suicide but also to natural death (Hawton *et al*., 2006). People who self-harm, therefore, constitute a particular fragile population needing special care. Overall, the Global Burden of Diseases study estimated that DSH ranks among the ten leading causes of disability-adjusted life years (DALYs) in people aged 15–39 worldwide (GBD_2015, 2016). The annual economic cost of DSH is high, reaching for instance $93 billion in total in the USA (Shepard *et al*., 2016), and 162 million pounds in England for general hospital costs only (Tsiachristas *et al*., 2017). Thus, an accurate estimation of the phenomenon is particularly important to assist organisation of care and prevention.

In the present study, we aimed to study mortality following DSH at a national level in order to limit potential biases related to local conditions. Indeed, while most previous studies were based on local data, very few used national registries (Payne *et al*., 2009; Runeson *et al*., 2010; Chung *et al*., 2012; Perry *et al*., 2012). To our knowledge, the largest cohort studies comprised around 40 000– 60 000 admissions or individuals in Scotland, Sweden, Ireland and Denmark (Payne *et al*., 2009; Runeson *et al*., 2010; Perry *et al*., 2012; Fedyszyn *et al*., 2016), and in Medicaid-financed adults in the USA (Olfson *et al*., 2017). Our database includes exhaustive information about approximately 45 million inhabitants in France covered by the general program of the public insurance system – i.e. 70% of the French population (the rest of the population being covered by specific insurance programs, whose data are currently not accessible) – over 2 consecutive years. The strength of this retrospective cohort is the very limited number of cases for whom the vital status is missing. The size of the sample allowed us to (i) examine DSH at a national scale in a country with an excellent universal insurance system; (ii) investigate the risk of short-term non-fatal DSH repetition; (iii) measure mortality rates at 1 year, in relation to suicide, accidents and natural causes; and (iv) weight risk factors including rare events like particular types of suicidal acts (drowning, gas, etc.). Regarding the high number of patients hospitalised for DSH each year in Western countries, we believe this study provides valuable information for clinicians working in Emergency departments, intensive care units or in liaison-consultation psychiatry for medical decision-making and to health institutions for care organization.

## Methods

### Population and database linkage

Data were extracted from two national registries. Medico-administrative data on all individuals hospitalised in medicine and surgery departments in France are routinely collected in a national database named ‘*Programme de Médicalisation des Systèmes d'Information*’ (*PMSI*). This database collects information about individuals’ name, date of birth, gender, place of residence, hospital location and diagnoses based on International Classification of Diseases– 10^th^ edition (ICD-10) codes. A second database named ‘*CepiDc’* monitored by the National Institute for Health Research (Inserm) provides individual information about date and cause of death, in addition to socio-demographic data (name, gender, date of birth) and geographical variables (place of death and place of residence). This information is based on death certificates completed by physicians and forensic institutes. The linkage of these two exhaustive databases is to date only possible for individuals under the general program of the national health insurance system, i.e. approximately 70% of the French population (45 million people in 2009). The process resulted from a recent collaboration between different administrations under a project named ‘Amphi’ in order to study post-hospitalisation mortality. This study was approved by the National Committee for data protection (registration number: 1454315). Patient written consent was not necessary for this study.

**Fig. 1.**
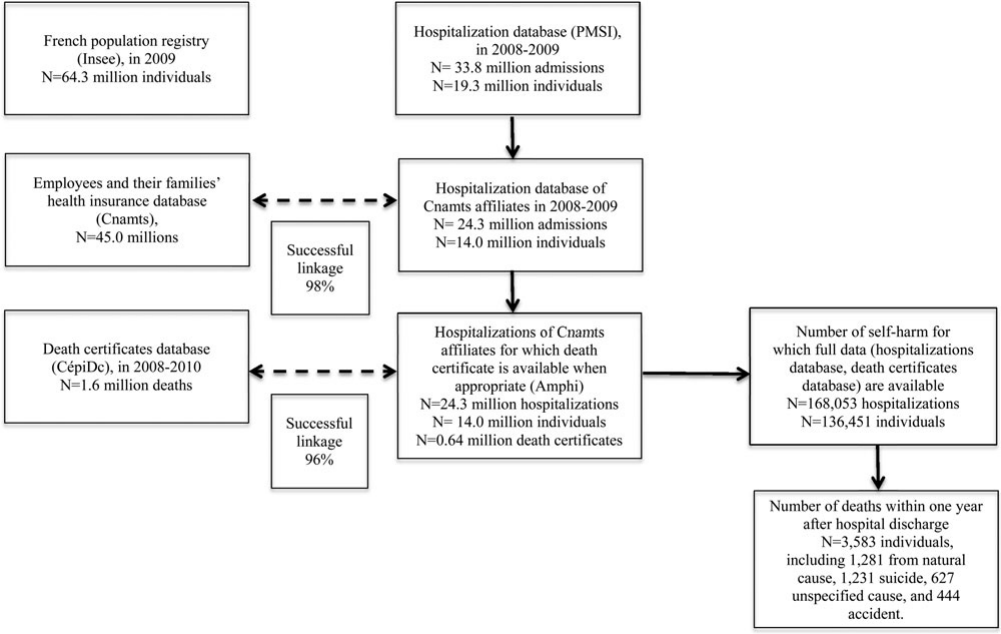
Flow chart representing the different steps of data collection.

We extracted data on individuals hospitalised for non-fatal DSH – corresponding to ICD-10 codes X60 to X84 – from 1 January 2008 to 31 December 2009. This extraction yielded 168 053 hospitalisations after exclusion of patients who died during the index hospitalisation, corresponding to 136 451 individuals and 0.7% of the total number of hospitalisations for the study population during this period. Based on probabilistic linkage according to different variables (the month and year of birth, full date of death, gender and residency location), mortality data could be obtained from CepiDc for 96% of the sample (see flow chart in Fig. 1). In order to investigate the representativeness of the data obtained from the Amphi database used in the current study in comparison with the whole PMSI database, we compared age and gender distributions in people who presented with DSH and found a very satisfactory overlap (data available on request).

*Footnotes:* Insee: Institut National de la Statistique et des Etudes Economiques; Cnamts: Caisse Nationale d’Assurance Maladie des Travailleurs Salariés; CepiDc: Centre d'Epidémiologie sur les causes médicales de décès; PMSI: Programme de Médicalisation des Systèmes d'Information; Amphi: Amphi database.

### Variables

We considered the first hospitalisation for DSH within the studied period as the index DSH episode. For each patient, socio-demographic and medical data were collected at the index hospitalisation: age, gender, type of hospital, distance from home to hospital, type of living place according to the zip-code, admission to intensive care unit, Charlson comorbidity index (Charlson *et al*., 1987), duration of hospital stay, transfer following initial care and means used for the index DSH episode. In the dataset, there were no missing data for all these variables except for zip-code (0.4% of missing data). Psychiatric diagnoses were not retained due to a high number of data missing (53% of individuals) and an expected lack of reliability in the way diagnoses were carried out in Emergency departments and medical/surgery facilities.

Some DSH means were pooled to facilitate analyses. The following categories were retained: intentional self-poisoning by drugs (X60 to 64), alcohol (X65), an unspecified chemicals or noxious substances (X66 and X68 to 69), gases and vapours (X67); intentional self-harm by hanging, strangulation or suffocation (X70), drowning and submersion (X71), firearm discharge (X72 to 74), use of explosive material, smoke, fire and flames, and steam, hot vapours and hot objects (X75 to 77), cutting with sharp or blunt objects (X78–79), jumping from a high place (X80), jumping or lying in front of moving objects or crashing of motor vehicle (X81–82), other specified means (X83) and unspecified means (X84). Violent means were all means except drugs and alcohol (X66 to 82).

Mortality and DSH recurrence were then examined within a 1-year period following the index DSH episode for each individual. Mortality data were collected for the period 2008–2010. Missing information on patient death 1 year after hospital discharge is only 0.1%. Causes of deaths were classified as suicide, accident, natural causes and undetermined causes.

### Statistical analyses

We present categorical variables as frequency distributions and continuous variables as means (with standard deviations, SD) or medians.

We developed logistic regression models to analyse the impact of factors associated with overall mortality and the four main causes of death, based on univariate analyses. In multivariate analyses, we introduced all individual variables considered significant in univariate analyses (*p* < 0.20) according to their clinical relevance based on correlation tests. We used backward selection. The overall accuracy of the model was assessed by the c-statistic, which represents the area under the curve (ROC). Finally, we analysed the impact of factors associated with a new hospitalisation for a recurrence of a DSH occurring within 1 year after the index hospitalisation. In order to analyse the potential time-dependent effect of recurrence of a DSH, we also used a Cox regression model including the same variables as above.

Proportional hazards assumption was checked for each variable of the Cox model, using graphs plotting the log of the negative log of the Kaplan-Meier survival function estimates *v*. log of the time to event, as well as including an interaction with log of the time to event in the model, considered as a sensitivity analysis. The proportional hazard assumption was satisfied for nearly all variables except for the duration of hospital stay. After taking into account this interaction in the final model, the hazard ratio estimates were almost identical to the results presented below. Only the effect of the duration of hospital stay was modified as longer durations tended to be associated with an increasing risk over time.

Although psychiatric diagnoses were not included as explicative variables in the main analyses for reasons exposed above, we performed a second sensitivity analysis with these variables in order to test the robustness of the model. This sensitivity analysis showed no significant change in the estimation of hazard ratios of the studied variables.

Finally, a Standardised Mortality Ratio (SMR) was calculated for each age group and gender as the ratio of death observed in the sample *v*. expected deaths in the general population.

## Results

### Description of the sample at index hospital admission

**Fig. 2.**
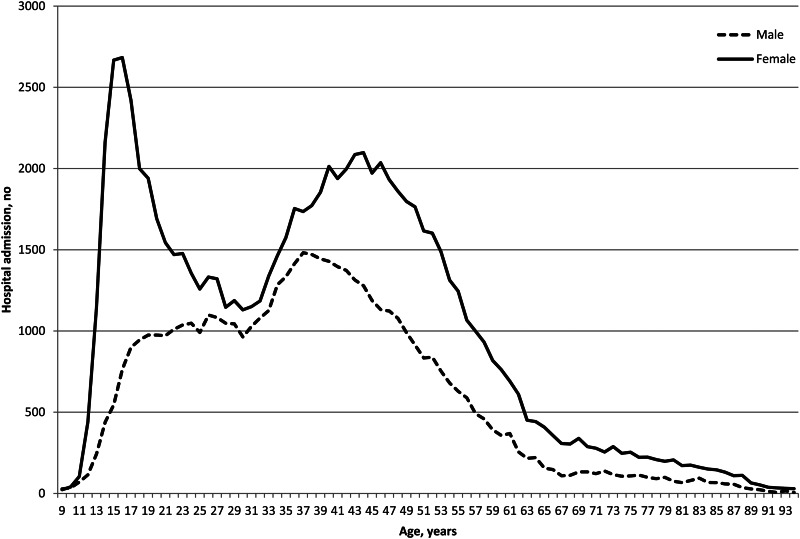
Distribution of age at index hospital admission for deliberate self-harm, by gender.

Description of the sample at the time of index admission can be found in Table 1 and distribution of hospital admissions for DSH is shown in Fig. 2.

**Table 1.** Description of the sample at the time of index admission

Variables	*N* = 136 451
Gender, female (%)	62.8
Age, median [min-max]	38 [9–94]
Age distribution (%):	
⩽25	26.0
26 to 64	67.9
⩾65	6.1
Type of living place (%):	
City centre	48.0
Suburbs	32.8
Small towns	10.6
Rural areas	8.6
Means used for index DSH (%)*:	
Drugs	82.1
Unspecified means	5.6
Alcohol ingestion	5.0
Cutting with sharp or blunt objects	4.9
Ingestion of unspecified chemicals or noxious substances	2.4
Hanging, strangulation or suffocation	1.6
Jumping from a high place	0.9
Firearm discharge	0.4
Other specified means	0.4
Drowning and submersion	0.3
Gases and vapors	0.3
Jumping or lying in front of moving objects or crashing of motor vehicles	0.2
Use of explosive material, smoke, fire and flames, and steam, hot vapours and hot objects	0.2
Use of a violent means (%)	11.2
Only means codes (%)	91.7
Type of index hospitalisation (%):	
General hospital	71.9
Academic hospital (CHU)	23.7
Private clinic	4.4
Admission to an intensive care unit (%)	12.9
Charlson Comorbidity index ⩾0	6.0
Mean Charlson index score when different from 0 (sd)	2.0 (1.4)
Duration of index hospital stay (%):	
<24 h	16.8
1 days	51.0
2–7 days	25.3
>7 days	6.9
Mean duration of hospital stay (sd)	2.4 (4.1)
Following hospitalisation (%):	
Discharged home	71.3
Transfer to psychiatric hospital in-patient care	23.7
Transfer to another medical or surgery department	5.0

Footnotes: *Some individuals are counted twice in case of use of multiple means.

### Recurrence of DSH within 1 year following index DSH

A majority of individuals were not re-hospitalised for a new DSH within the following year as recurrence was found in only 12.4% (*N* =  16 919) of the sample. Median delay between the index act and the following one was 84 days. The first recurrence happened within the first week in 11.4% of people who re-presented, within the first month in 28.0%, within the first 3 months in 52.4%, and within the first 6 months in 75.2%. Most individuals used the same means between the index act and the next DSH (78.1%), while 6.6% switched from a non-violent to a violent means and 5.1% from a violent to a non-violent means (unspecified in 10.2%).

Most DSH recurrence occurred only once within the 1-year follow-up (*N*  =  12 616, 74.6%) while 16.1% re-presented a second time, and 9.3% more than 3 times, including 2.1% more than 5 times.

### One-year mortality following DSH

**Fig. 3.**
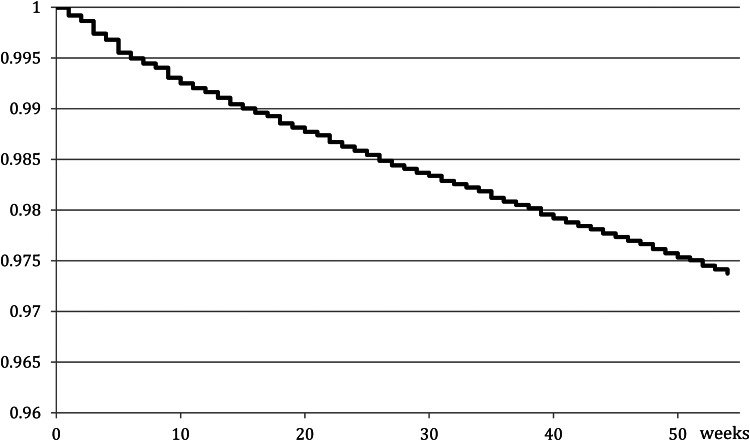
Global survival curve since index hospital admission for deliberate self-harm.

The death occurred in 2.6% (*N*  =  3583) of the total sample within 1 year following the index DSH episode. Figure 3 shows the global survival curve over the 1-year follow-up. The SMR relative to expected deaths in the general population was 8.5 in men (from 3.3 in those aged 76 and above, to 24.5 in 26–35 s), 6.6 in women (from 2.8 in 76 + to 28.9 in 26–35 s) and 7.5 overall.

Causes of deaths were: natural causes (35.7% of death cases), suicide (34.4%), unspecified (17.5%) and accident (12.4%). Among men, 3.9% had died within 1 year, including 1.4% due to suicide and 1.3% due to natural causes. The equivalent figures for women were 1.9, 0.6 and 0.7%, respectively. Figure 1 in supplemental material represents the number of deaths per age group and gender for each cause.

The means used for suicide were hanging, strangulation or suffocation in 42.1%, drugs in 28.4%, jumping from a height in 6.3%, drowning and submersion 5.4%, firearm discharge 5.0%, ingestion of unspecified chemicals or noxious substances 2.5%, jumping or lying in front of moving objects or crashing of motor vehicle 1.7%, cutting with sharp or blunt objects 1.5%, use of explosive material, smoke, fire and flames, steam 1.1%, self-poisoning by gases and vapours 0.7%, ingestion of alcohol 0.1%, other specified means 0.3% and unspecified means 4.9%.

Overall concordance of suicidal means between the index DSH and suicide was 30.2% but with large differences according to the means used: 79.9% of those who died by suicide using drugs previously used the same methods for the index DSH, while the proportion was 35.5% for chemicals, 22.6% for firearm, 15.8% for cutting with sharp or blunt objects, 9.8% for hanging and below 8% for all other means. It is important to note that the majority of individuals who died by suicide had used drug overdose at the index DSH (*N*  =  878 among 1231 suicide deaths, 71.3%).

Death by suicide occurred within the first week following the index DSH in 5.5%, within the first month in 20.5%, within the first 3 months in 41.7%, and within the first 6 months in 62.9%. The cumulative risk for the other causes of death showed a more linear distribution over the 1-year follow-up (Fig. 2 in Supplemental material).

### Predictive factors for 1-year mortality

Tables 2 and 3 present the results of logistic regression analyses for overall mortality and the four main causes of death. While violent suicidal means at the index DSH index and admission to an intensive care unit were both significantly associated with future suicide, their positive predictive values were low (1.6% and 1.6%, respectively). However, the global model including all factors of the regression analysis yielded a fair-good accuracy with an area under the curve of 0.77.

**Table 2.** Risk factors for overall mortality and suicide

	All causes				Suicide			
Variables	Hazard ratio	95% CI		*p*	Hazard ratio	95% CI		*p*
Age (reference = 9–15 year-old)								
16–25	4.0	2.0	7.9	<0.0001	3.6	1.5	9.0	0.005
26–35	11.9	6.1	23.1	<0.0001	8.3	3.4	20.4	<0.0001
36–45	16.3	8.4	31.6	<0.0001	12.2	5.0	29.5	<0.0001
46–55	22.9	11.9	44.2	<0.0001	17.0	7.0	41.2	<0.0001
56–65	32.6	16.8	63.0	<0.0001	19.0	7.8	46.3	<0.0001
66–75	49.8	25.7	96.7	<0.0001	28.0	11.3	69.1	<0.0001
76 +	92.0	47.6	178.2	<0.0001	29.3	11.8	72.9	<0.0001
Gender: Male*v.* female	2.0	1.9	2.1	<0.0001	2.1	1.9	2.4	<0.0001
Means used for index DSH (ref = drugs [X60-X64])								
Violent means [X66-X82]	1.4	1.2	1.5	<0.0001	1.5	1.3	1.8	<0.0001
Alcohol [X65]	1.1	1.0	1.3	0.2	0.4	0.3	0.7	<0.0001
More than one DSH means (*v*. single)	0.9	0.7	1.0	0.1	1.3	1.0	1.7	0.1
University *v*. general hospital	1.0	1.0	1.0	0.4	0.8	0.7	0.9	0.002
Private clinic *v*. general hospital	0.9	0.7	1.0	0.06	0.9	0.7	1.2	0.5
Distance from home to hospital (ref = 0–3 km)								
4–12 km	0.9	0.9	1.0	0.2	1.1	0.9	1.3	0.2
13 km and more	0.9	0.9	1.0	0.2	1.1	0.9	1.2	0.4
Living place (ref = city centre)								
Suburbs	1.0	0.9	1.0	0.4	0.9	0.8	1.0	0.05
Small town	1.0	0.9	1.1	0.9	1.0	0.9	1.3	0.5
Rural	0.8	0.7	1.0	0.01	0.8	0.7	1.0	0.1
Admission to intensive care unit (yes *v*. no)	1.2	1.1	1.3	<0.0001	1.5	1.3	1.7	<0.0001
Comorbidity (ref : Charlson index = 0)								
Charlson = 1–2	1.6	1.4	1.7	<0.0001	0.8	0.6	1.0	0.02
Charlson = 3–4	1.9	1.6	2.3	<0.0001	0.4	0.2	0.8	0.01
Charlson = 5 and more	5.5	4.6	6.5	<0.0001	1.3	0.7	2.5	0.4
Duration of hospital stay (ref = 0–1 night)								
2–3 nights	1.1	1.0	1.2	0.06	1.2	1.0	1.4	0.03
4 and more nights	1.1	1.0	1.2	0.1	1.1	0.9	1.3	0.4
Transfer following initial care (ref = home return)								
to another medical department	2.0	1.8	2.2	<0.0001	2.3	1.9	2.8	<0.0001
to a psychiatric inpatient care unit	1.2	1.1	1.3	<0.0001	1.7	1.5	1.9	<0.0001

**Table 3.** Risk factors for death from natural causes, accident and unspecified cause

	Accident				Natural causes				Unspecified cause			
Variables	Hazard ratio	95% CI		*p*	Hazard ratio	95% CI		*p*	Hazard ratio	95% CI		*p*
Age (ref = 9–15 year-old)												
16–25	2.8	0.7	11.9	0.2	3.7	0.5	28.6	0.2	6.8	0.9	49.9	0.06
26–35	9.4	2.3	38.4	0.002	15.2	2.1	110.5	0.007	25.4	3.5	182.5	0.001
36–45	9.3	2.3	37.8	0.002	34.0	4.8	243.0	0.0004	30.5	4.3	218.8	0.0007
46–55	8.9	2.2	36.3	0.002	64.7	9.1	461.5	<0.0001	39.7	5.5	284.1	0.0002
56–65	14.2	3.5	58.5	0.0002	127.7	17.9	910.9	<0.0001	46.1	6.4	332.3	0.0001
66–75	13.1	3.1	56.5	0.0005	226.9	31.8	1620.1	<0.0001	58.3	8.0	425.6	<0.0001
76 +	42.7	10.3	176.3	<0.0001	449.0	63.0	3199.9	<0.0001	139.0	19.2	1003.1	<0.0001
Gender: Male vs. female	2.2	1.8	2.7	<0.0001	1.829	1.6	2.1	<0.0001	2.0	1.7	2.4	<0.0001
Means used for index DSH (ref = drugs [X60-X64])												
Violent means [X66-X82]	1.2	0.9	1.5	0.3	1.3	1.1	1.5	0.001	1.3	1.0	1.7	0.02
Alcohol [X65]	1.7	1.1	2.5	0.01	1.4	1.2	1.8	0.001	1.3	0.9	1.9	0.1
More than one DSH means (*v*. single)	0.7	0.4	1.1	0.1	0.8	0.6	1.1	0.2	0.7	0.4	1.1	0.1
University *v*. general hospital	0.9	0.7	1.1	0.4	0.9	0.8	1.1	0.3	1.5	1.2	1.8	<0.0001
Private clinic *v*. general hospital	0.4	0.2	0.8	0.006	0.9	0.7	1.2	0.4	0.9	0.6	1.4	0.8
Distance from home to hospital (ref = 0–3 km)												
4–12 km	0.8	0.6	1.1	0.1	1.0	0.8	1.1	0.5	0.8	0.6	0.9	0.009
13 km and more	0.9	0.7	1.2	0.6	0.9	0.8	1.1	0.3	0.8	0.6	1.0	0.03
Living place (ref = city centre)												
Suburbs	1.1	0.9	1.4	0.2	1.0	0.8	1.1	0.6	1.1	0.9	1.3	0.5
Small town	1.0	0.7	1.4	0.9	1.0	0.8	1.2	0.6	0.8	0.6	1.1	0.2
Rural	0.8	0.5	1.2	0.2	1.0	0.7	1.1	0.3	0.7	0.5	1.0	0.04
Admission to intensive care unit (yes *v*. no)	1.9	1.4	2.4	<0.0001	0.9	0.8	1.1	0.2	1.4	1.1	1.7	0.003
Comorbidity (ref : Charlson index = 0)												
Charlson = 1–2	1.4	1.0	1.9	0.06	2.7	2.4	3.2	<0.0001	1.3	1.0	1.7	0.08
Charlson = 3–4	2.1	1.2	3.7	0.009	3.9	3.1	4.8	<0.0001	0.6	0.3	1.3	0.2
Charlson = 5+	1.6	0.6	4.5	0.3	13.7	11.1	16.9	<0.0001	3.5	2.1	5.8	<0.0001
Duration of hospital stay (ref = 0–1 night)												
2–3 nights	0.8	0.6	1.1	0.2	1.2	1.0	1.5	0.01	0.8	0.7	1.1	0.1
4 and more nights	0.7	0.5	0.9	0.008	1.2	1.0	1.4	0.04	1.0	0.8	1.3	0.7
Transfer following initial care (ref = home return)												
to another medical department	2.4	1.8	3.2	<0.0001	1.9	1.6	2.1	<0.0001	1.6	1.2	2.0	0.001
to a psychiatric inpatient care unit	1.3	1.0	1.6	0.03	0.8	0.7	1.0	0.02	1.1	0.9	1.3	0.2

Cox regression model showed that recurrence of a DSH was also associated with an increased risk of overall mortality (HR  =  1.98 [1.78–2.21]) and was related to suicide (HR  =  2.30 [1.92–2.75]) but also accident (HR  =  2.70 [2.06–3.55]), natural causes (HR  =  1.44 [1.15–1.79]) and unspecified causes (HR  =  2.15 [1.68–2.75]).

Detailed results according to DSH means are presented in Supplemental material.

## Discussion

The present study is the largest one published to date in terms of a number of individuals followed over 1 year after a DSH and for whom mortality data are available. Other strengths of this sample are the extent of the national coverage (almost 70% of the French population), the totally exhaustive data collection of hospitalisations and the very limited number of cases for which vital status was not known after 1 year (4%). Main results of this study are: (1) At index admission, individuals who self-harmed were mainly women (63%) with a median age of 38 and a narrow peak at 16, using medication overdose (82.1%). Most (71.3%) returned home the same day or after a short stay at the hospital (1 day or less for almost 70%). (2) Recurrence of DSH within 1 year occurred in 12.4% of cases, during the first 6 months in 75.2% of cases and usually once only. (3) Mortality at 1 year reached 2.6% of the sample, shared between suicide and other causes. In 63% of cases, suicide occurred within the first 6 months. Classical risk factors were identified but were of extremely limited predictive value.

Several limitations have to be highlighted before discussing findings. First, data for about 30% of the French population were not available, limiting representativeness. This concerns individuals with specific jobs – which currently benefit from special insurance programs – notably farmers, self-employed workers, most students, or soldiers. While the comparison of the distributions of age and gender in individuals with DSH affiliated or not to the general program did not show significant differences, the modulating effect of specific professional groups on particular variables, especially suicide risk, cannot be excluded. Second, while individuals who engage in DSH acts are usually referred to an emergency department for physical examination and the most severe acts are hospitalised even for a few hours, an unknown number of DSH episodes are not. This includes patients directly referred to a psychiatric hospital and those not hospitalised. Moreover, the identification of cases with DSH solely relies on the coding made by hospital physicians. These factors lead to an underestimation of the DSH phenomenon only based on hospital reports, as previously shown (Geulayov *et al*., 2018). Third, the misclassification of causes and undetermined causes are major issues in suicide research (Kapusta *et al*., 2011). For instance, a study in England showed that only 65% of suicide cases as determined by researchers had been given a suicide verdict by coroners, and coroners were less likely to give suicide verdicts in cases of poisoning, drowning and jumping (Palmer *et al*., 2015). In our study, this could explain a strong association between DSH by drowning and future accidental death but not suicide, or the lack of association between DSH by jumping and future suicide. Fourth, no information is currently available in administrative databases on particular known suicide risk factors, such as suicidal intent, personality traits, a history of childhood abuse, recent life events, among others. Also, some data could not be retained for analyses. This is notably the case for psychiatric diagnoses regarding their expected lack of reliability from emergency units and other medico-surgery facilities. Furthermore, there is a lack of current access to potential outpatient psychiatric care during the 1-year follow-up. Moreover, we had no information about a history of DSH before the index admission. Index admission in the current study is therefore not synonymous as a personal history of first hospitalisation for DSH. Finally, 2008–2009 was the only period for which full data were available with a follow-up of 1 year at the time of analyses. More data will be available in the next years. Of note, recent reductions in DSH and suicide rates in France suggest that prevalence rates are currently lower. Keeping in mind these limitations, our study yielded or confirmed several valuable findings at a large scale.

An important number of individuals were hospitalised for DSH in France in 2008 and 2009. Our sample was largely similar to previous studies conducted in different countries (Hawton *et al*., 2015) with almost 63% of women, and 50% were aged below 38 years (including 26% below 25 years). There was a notable peak in females aged 16 years. Almost 80% of individuals used self-poisoning with medication at the index act, again in line with previous studies (Runeson *et al*., 2016). In most cases, hospital stays were of short duration (almost 70% staying one night or less; 79.4% less than 1 week in intensive care) and 70% returned home. These observations raise several questions, notably (i) the relevance of developing specific programs targeting adolescents and young adults to prevent initial acts; (ii) the organization and feasibility of care programs offered at hospital discharge in light of a large number of individuals engaging in DSH episodes. This latter point should also take into account the recognised fact that many individuals do not attend medical appointments after DSH (Haw *et al*., 2002), necessitating pro-active interventions.

A recurrence of DSH hospitalisation occurred in 12.4% of the sample within 1 year, during the first 6 months in 75% of cases (suggesting a same unresolved crisis), and usually only once (75%). The rate of recurrence in our study was slightly lower than reported by one meta-analysis (16.3% (CI 15.1–17.7) (Carroll *et al*., 2014)). Most recurrent acts involved a similar means as the index act, mostly drug overdose, as previously reported (Owens *et al*., 2015).

After 1 year, 2.6% of the total sample had died. Our study confirmed that people who engage in DSH are a fragile population related to premature mortality in comparison with the general population, with an overall SMR of 7.5. SMRs even reached more than 20 in specific age groups, notably young adults. Importantly, the main causes of death were shared between suicide, natural causes and accident as previously reported (Hawton *et al*., 2006; Karasouli *et al*., 2011; Bergen *et al*., 2012*b*). Follow-up of patients with DSH should therefore include both somatic and psychiatric care in a holistic approach.

Suicide was the cause of death for 0.9% of the DSH sample, a result lower than the one reported by a recent meta-analysis (1.6% (CI 1.2–2.4%) (Carroll *et al*., 2014)). The same study however noted large between-study variations in 1-year mortality rates and our findings are within the same range as several previous studies (Hawton *et al*., 2003; Bergen *et al*., 2012*b*).

Almost 63% of suicides occurred within the first 6 months following DSH, a previously highlighted critical period (Cooper *et al*., 2005). While the risk of suicide may persist for years in some individuals who attempt suicide (Suominen *et al*., 2004; Hawton *et al*., 2015), follow-up studies have previously shown that this initial period is critical regarding mortality risk following DSH (Hawton *et al*., 2015). This period may therefore represent both the most sensitive but also convenient time frame to prevent suicidal behaviour, including suicide death and DSH recurrence.

Risk factors at the time of DSH for subsequent mortality and suicide were similar as previous studies: older age, being male, use of a violent DSH means, being admitted to an intensive care unit, recurrence of DSH and being transferred to another medical department or to a psychiatric inpatient care unit. Among violent DSH, hanging/strangulation/suffocation, use of a sharp object, firearm, gases and chemical or noxious substances were all significantly associated with an increased risk of future suicide, although significant associations with other violent means (e.g. jumping from height, drowning, immolation, jumping in front of a vehicle) may not have been found due to a lack of power, frequent death before or during the index hospitalization or, again, misclassification. The supplementary suicide risk conferred by a violent *v*. non-violent methods (mainly drug overdose) has previously been reported (Bergen *et al*., 2012*a*; Stenbacka and Jokinen, 2015; Olfson *et al*., 2017; Beckman *et al*., 2018).

In our sample, a little less than a quarter of patients were admitted in a psychiatry inpatient unit after the index act, which is higher than in some countries (e.g. 7% in the UK (Cooper *et al*., 2013)). Admission to psychiatry likely reflects concerns by the physician about the level of short-term suicide risk or the severity of comorbid psychopathology but also available local beds and outpatient facilities. It is noteworthy that psychiatry inpatient care was not associated with a lower risk of suicide death compared with individuals who also engaged in DSH but were not hospitalised in psychiatry. These results are in line with previous studies in other countries (Hjorthøj *et al*., 2014; Kapur *et al*., 2015). We can only speculate on the reasons such as more severe psychopathology, inadequate care with regard to suicide risk in some psychiatric hospitals, and insufficient follow-up after hospitalisation. Discharge from psychiatric hospital is associated with a short-term risk of suicide, notably within the first 3 months (Olfson *et al*., 2016).

Studies have shown that clinician's estimate of suicide risk is insufficient. In the present study, we confirmed several risk factors for suicide. However, if taken alone, reported risk factors are of little help at the time of index hospitalisation to identify the individuals who will die from suicide within the next year. Indeed, positive predictive values for use of violent suicidal means or admission in intensive care unit hardly reached 2%. i.e. less than 2% with the risk factor will die from suicide. Similar results were previously reported for the use of scales alone (Carter *et al*., 2017), leading governmental agencies like NICE in England (NICE_guidelines, 2011) to propose risk reduction to all patients following DSH.

In conclusion, this national study confirmed that people who engage in DSH are at increased risk of both short-term DSH recurrence and premature death from suicide, but also other causes. The critical period appears to be the first 6 months following DSH. Individual risk factors are of very limited utility suggesting that all patients should be followed in the aftercare of DSH as long as more specific risk factors (or combination of factors) are not identified. These results support currently developing strategies to reduce both DSH recurrence and related mortality.

## Supplementary material

For supplementary material accompanying this paper visit http://dx.doi.org/10.1017/S2045796019000039. click here to view supplementary material

## Availability of data and materials

Data cannot be shared to date as they include administrative non-anonymous information that can only be handed by selected institutions (here, the Ministry of Health).
