# Supplementary material for: Recurrence and mortality 1 year after hospital admission for non-fatal self-harm: a nationwide population-based study
Source: Epidemiol Psychiatr Sci. 2019 Feb 18;29:e20. doi: 10.1017/S2045796019000039 (PMC8061131; doi:10.1017/S2045796019000039)
Supplement: Supplementary file 1 [file S2045796019000039sup001.docx]

**SUPPLEMENTAL MATERIAL**

**Figure 1. Number of deaths per age group and gender for each of the 4 mortality causes during the one-year period following hospitalization for deliberate self-harm.**

**Figure 2. Cumulative risk curves for specific causes of mortality since index hospital admission for deliberate self-harm.**

**Predictive risk factors of mortality according to DSH means**

Detailed examination according to DSH means showed an increased risk of overall mortality at one year in comparison to drug ingestion when the index DSH method was hanging, strangulation or suffocation (OR=1.75 95%CI [1.45-2.12]), including in relation to suicide (OR=2.05 [1.55-2.70]) and natural causes (OR=1.92 [1.41-2.62]); and cutting with sharp or blunt objects (OR=1.39 [1.19-1.62]) in relation to suicide (OR=1.35 [1.05-1.73], natural causes (OR=1.37 [1.07-1.78]) and unspecified causes (OR=1.60 [1.13-2.25]). Moreover, firearm discharge (OR=1.99 [1.23-3.20]), gases and vapors (OR=2.32 [1.30-4.13]) and ingestion of unspecified chemicals and noxious substances (OR=1.39 [1.02-1.90]) were all associated with an increased risk of future suicide, while drowning and submersion was associated with future accidental death only (OR=2.70 [1.10-6.65]). Surprisingly, use of unspecified means at index DSH tended to be associated with increased risks of death by unspecified causes one year later (OR=1.5 [1.1-2.0]). Other means (jumping from height, jumping in front of a vehicle, immolation/explosion) were not significantly associated with increased one-year mortality relative to drug ingestion.
